# Sensory defects and developmental delay among children with congenital rubella syndrome

**DOI:** 10.1038/srep46483

**Published:** 2017-04-13

**Authors:** Michiko Toizumi, Giang Thi Huong Nguyen, Hideki Motomura, Thanh Huu Nguyen, Enga Pham, Ken-ichi Kaneko, Masafumi Uematsu, Hien Anh Thi Nguyen, Duc Anh Dang, Masahiro Hashizume, Lay-Myint Yoshida, Hiroyuki Moriuchi

**Affiliations:** 1 Department of Pediatric Infectious Diseases, Institute of Tropical Medicine, Nagasaki University, Nagasaki, Japan; 2 Rehabilitation Department, National Pediatrics Hospital, Hanoi, Vietnam; 3 Department of Pediatrics, National Hospital Nagasaki Medical Center, Omura, Japan; 4 Department of Pediatrics, Khanh Hoa General Hospital, Nha Trang, Vietnam; 5 Department of Otolaryngology, Nagasaki University Hospital, Nagasaki, Japan; 6 Department of Ophthalmology, Nagasaki University Hospital, Nagasaki, Japan; 7 Department of Bacteriology, National Institute of Hygiene and Epidemiology, Hanoi, Vietnam; 8 Department of Pediatrics, Nagasaki University Hospital, Nagasaki, Japan; 9 Graduate School of Biomedical Sciences, Nagasaki University, Nagasaki, Japan

## Abstract

This study investigated the features of developmental difficulties combined with sensory defects in children with congenital rubella syndrome (CRS). Following a large rubella outbreak in Khanh Hoa Province, Vietnam, in 2011, we enrolled 41 children with CRS from September 2011 through May 2013. Fourteen participants died and six became untraceable by October 2013; the remaining 21 children were followed up from 2013 to 2015. Thirteen and seven participants had hearing and functional ophthalmological impairment, respectively. Developmental difficulties were suspected in 19 (95%) children who failed in at least one area of the Ages and Stages Questionnaire (ASQ) and/or Denver II in 2013 and/or 2015. Developmental difficulties were frequently identified in the ASQ communication domain (n = 14 in 2013) and Denver II language area (n = 13 in 2013). Seven (41%) participants were suspected of having autism spectrum disorder (ASD) in 2013 by the Modified Checklist for Autism in Toddlers. In 2015, proportions of children failing the problem-solving (62%) and personal–social (62%) domains had increased and two of 13 were diagnosed with ASD by the Childhood Autism Rating Scale, Second Edition. Developmental difficulties were suspected in most children with CRS, including autism largely combined with sensory dysfunction.

Rubella is usually a self-limited illness; however, rubella infection during early pregnancy can result in miscarriage, fetal death or the combination of disabilities known as congenital rubella syndrome (CRS), characterized by sensorineural hearing impairment, cataracts, cardiac defects, and/or damage to the brain and nervous system^1^,^2^. After the widespread emergence of CRS in the United States during the 1960s, intellectual disability and autism were found in about 42% and 7.4% of CRS patients, respectively, which are much higher than the proportions found in the general population^3^,^4^,^5^.

The introduction of rubella-containing vaccines (RCVs) has led to a sharp decrease of CRS in countries where screening, diagnosis, and intervention for developmental and sensory difficulties in young children are widely available^6^. Thus, there are few comprehensive developmental assessment studies of CRS patients with recently established and currently used screening or assessment tools.

During an enrollment phase (from May 2009 to May 2010) of a birth cohort study in Khanh Hoa Province, Vietnam, we found that 29% (95% confidence interval, 27–31%) of enrolled pregnant women were susceptible to rubella^7^. The following year, a large-scale rubella outbreak occurred throughout Vietnam between January and July 2011^8^ and many CRS cases emerged. To characterize the clinical manifestations of CRS, we studied infants with CRS, focusing especially on cardiovascular defects; we found high mortality and an association between pulmonary hypertension and death^9^. In the present study, we followed up the children with CRS and assessed their developmental, ophthalmological, and otological status using current screening or assessment tools.

## Results

### Ophthalmological defects and hearing impairment among children with CRS

**Figure 1:**
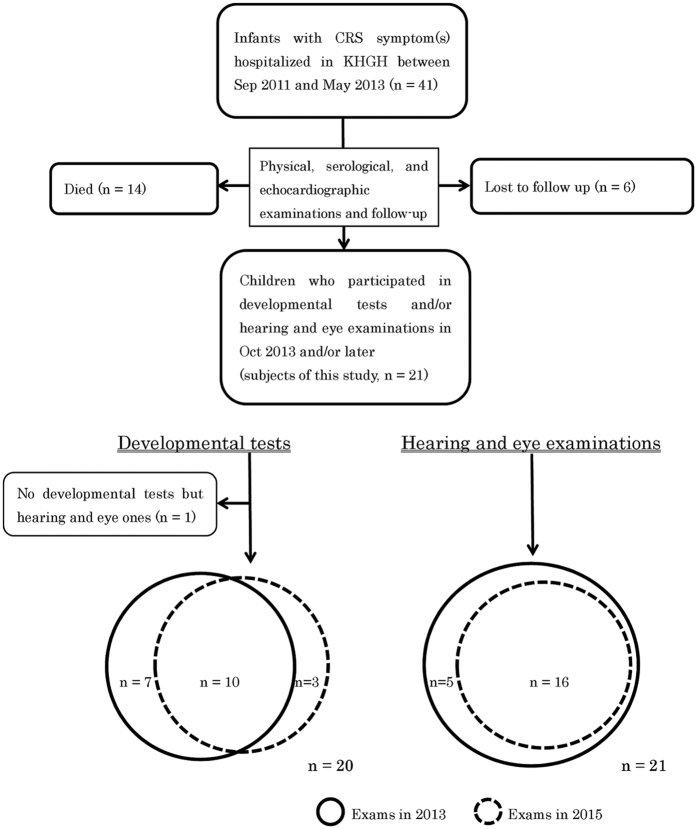
Flowchart of study participant selection and the number of children evaluated by developmental testing in 2013 and 2015 and ophthalmological and otological testing in 2013 and 2015.

A total of 41 children with CRS were enrolled in the study. By the October 2013 follow-up, 14 had died and six were lost to follow-up. The remaining 21 participants, including 12 girls and 9 boys, underwent ophthalmological and otoscopic examination and automated auditory brainstem response (AABR) tests in October 2013 (median age 23.0 months, interquartile range (IQR) 1.5). Sixteen children had the same examinations and an additional test for visual acuity in October 2015 (n = 16, median age 46.7 months, IQR 1.8) (Fig. 1).

On follow-up examination in 2013 and 2015, we found ophthalmological abnormalities in 11 (52%) of the 21 (Tables 1 and 2) children. The most frequent abnormality was pigmentary retinopathy (n = 10, 48%), which is one of the specific findings among CRS patients. Seven participants (33%) had other ocular abnormalities such as cataract, myopia, hyperopia, strabismus, microphthalmia, and nystagmus. Cataract was present in four children (19%); one was unilateral and three were bilateral. Three participants had already undergone cataract surgery by 2013 but the remaining case of bilateral cataracts had not, even by 2015 (ID 11). All participants with cataracts also had microphthalmia and strabismus. The intraocular pressure was less than 21 mmHg in all cases, except for one who presented with high intraocular pressure (24 mmHg) in the right eye. However, this child was crying during the examination and had no glaucomatous change in the optic discs. Therefore, we considered that none of the participants had glaucoma. Among children who underwent a visual acuity test in 2015, five had 1.0 vision in both eyes and one (ID 21) had 0.6 and 0.7 in the right and left eyes, respectively, although he could not complete the test owing to a lack of cooperation. One child (ID 11) with untreated bilateral cataracts could detect hand motion in both eyes and the right eye and had light perception in the left eye. None of the nine participants with hearing impairment could perform the visual acuity test. Two children who had undergone bilateral cataract surgery wore glasses; another child with an intraocular lens after unilateral cataract surgery did not wear glasses. Glasses had been recommended for one girl (ID 12) with hyperopia in 2013; however, she did not yet wear glasses in 2015.

Among the 21 children evaluated in 2013 for hearing ability using the AABR test, 13 (62%) were suspected of being hearing impaired. Nine of them had moderate or greater bilateral hearing impairment, defined as an absence of response to 45 dB in both ears. One child had undergone bilateral cochlear implantation at the time of follow-up in 2013; therefore, a total of 10 children were regarded as having moderate or greater bilateral hearing impairment (Table 1). Otoscopic examination revealed that three participants had bilateral and two had unilateral otitis media with effusion and/or retraction of the tympanic membrane (Table 1). One child (ID 14) had cleft lip and palate for which he had undergone surgery, a fistula in the right auricle and a cleft right earlobe. Results of AABR testing in 2015 (n = 16) were the same as in 2013, except for one child who could not complete the examination. The otitis media with effusion and/or retraction of the tympanic membrane in five cases had cleared up by 2015. None of the children had used hearing aids in 2013; in 2015, one child had begun to use a hearing aid in both ears but they seemed ineffective in improving his hearing ability (ID 5). One boy with bilateral cochlear implants could speak fluently (ID 19). Only one child with suspected bilateral hearing impairment attended a school for children with special needs in Khanh Hoa Province (ID 3).

### Overall developmental and sensory features of children with CRS

A total of 20 children were evaluated for developmental features, 17 (median age 24.7 months, IQR 1.5) in December 2013 and 13 (median age 43.5 months, IQR 1.5) in July 2015, 10 for the second time and 3 for the first time (Fig. 1 and Table 1).

Nineteen of the 20 children (95%) who had undergone developmental testing in 2013 and/or 2015 were suspected of having developmental difficulties, with an “abnormal” score in at least one domain of the Ages and Stages Questionnaire, Second Edition (ASQ)^10^,^11^ or a “suspect” score in at least one area of the Denver Developmental Screening Test II (Denver II)^12^,^13^ in 2013 and/or 2015. Among the 19 children suspected of having developmental difficulties, hearing impairment was suspected in 11 (58%) and 6 (37%) also had one or more functional ophthalmological problems. In addition to the above 20 children, we assessed developmental milestones in one child (ID20) who could not complete all the developmental assessment sessions and found signs of developmental delay. This child had begun holding their head up at 14 months of age and did not yet sit up at 22 months of age.

### Developmental status of children with CRS using screening tests

**Figure 2:**
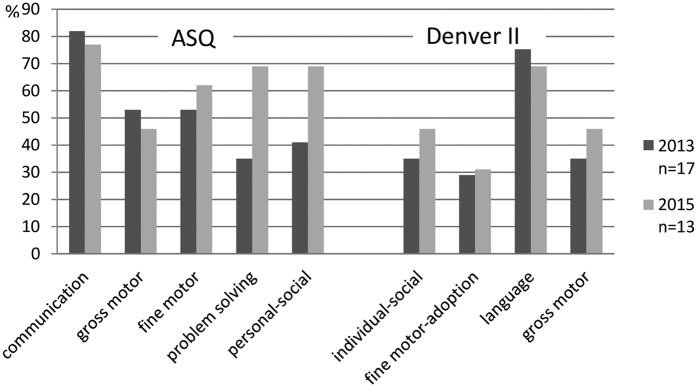
Proportion of children who failed each domain and area in the Ages and Stages Questionnaire and Denver II in 2013 and 2015.

In 2013, “abnormal” ASQ results were obtained among participants, particularly for the communication domain (82%), and “suspect” Denver II results were mostly found in the language area (76%). In 2015, the proportion of children who failed in the communication domain of ASQ and language area of the Denver II remained high (85% and 69%, respectively). The proportion of participants who failed in the ASQ problem-solving and personal–social domains had increased (69% and 69%, respectively) (Fig. 2). To assess developmental progress in these children, we compared the ASQ results of 10 children who underwent testing in both 2013 and 2015. We found three children who passed the first round of testing but failed the next round in the problem-solving domain; another three children passed the first round but failed the subsequent round in the personal–social domain (Table 1).

### Quantitative comparison of ASQ scores

**Figure 3:**
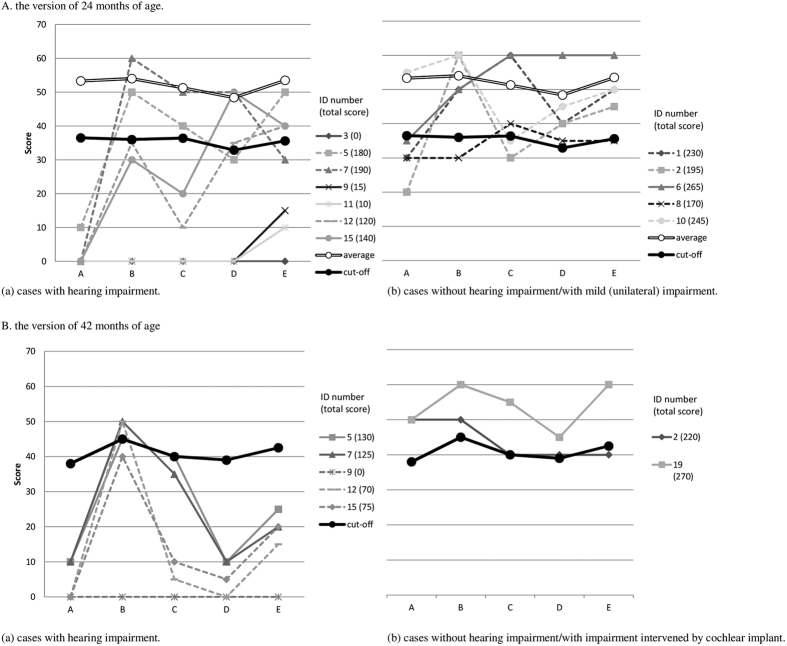
Participant scores in each domain of the Ages and Stages Questionnaire (ASQ), versions for 24 and 42 months of age. Solid lines: Children with functional ophthalmological disorders. Dotted lines: Children without functional ophthalmological disorders. Average: mean score in each domain of ASQ for 1,494 Vietnamese children aged 24 months. ASQ domains: A, communication; B, gross motor; C, fine motor; D, problem-solving; E, personal–social.

To analyse the severity of developmental delay, scores for each ASQ domain were compared among the 12 children using the 24-months version of the ASQ (Fig. 3A). We used the mean (standard deviation) of scores for the communication, gross motor, fine motor, problem-solving, and personal–social ASQ domains, calculated from the data of 1,494 Vietnamese children who were 24 months old. Average scores for these domains among the children were 53.3 (11.7), 54.0 (7.5), 51.3 (9.6), 48.4 (8.8), and 53.3 (7.7), respectively. We found varying levels of severity and a tendency for children with hearing impairment to have severer developmental delay (Fig. 3A(a) vs. (b)). We compared scores for each ASQ domain among seven children using the 42-months version of the ASQ (Fig. 3B; results for six of them are also shown in Fig. 3A). One child with no hearing loss and another who had a cochlear implant obtained scores around or above the cut-off values (Fig. 3B(b)).

The other children with bilateral hearing loss had much lower scores, especially in the communication, problem-solving, and personal–social domains (Fig. 3B(a)).

### Autism spectrum disorder

Seventeen children who failed the ASQ or Denver II were evaluated using the Modified Checklist for Autism in Toddlers (M-CHAT)^14^ in 2013, and seven (41%) of them were suspected of having autism spectrum disorders (ASD) (Tables 1 and 3). An experienced paediatric neurologist (G.T.H.N.) examined the children and diagnosed four cases (IDs 2, 7, 12, and 14) as non-ASD based on their good eye contact, good response to non-verbal approach, and lack of any abnormal behaviour or disinterest. The remaining three participants (IDs 3, 9, and 11) were further checked according to Diagnostic and Statistical Manual of Mental Disorders IV (DSM-IV) criteria for autistic disorder; two (IDs 9 and 11) were classified as having autistic disorder. Six of the seven children had hearing impairment and five had a functional ophthalmological disorder. Five participants had a score under the ASQ cut-off value in the gross motor domain, and six scored under the cut-off value in the fine motor domain.

In 2015, we assessed 12 children for ASD using the Childhood Autism Rating Scale, Second Edition (CARS2)^15^, including 6 suspected of having ASD by M-CHAT. One boy who did not fail in any areas of the ASQ or Denver II was not evaluated. The median total raw score of CARS2 was 24.3 (IQR 8.5). Two girls were diagnosed as having ASD (score ≥30) and both were categorized as having severe ASD (≥37). One girl (ID 9), aged 44 months, had been diagnosed in 2013 with ASD. In 2015, her CARS2 score was 40.5, and she failed all domains of the ASQ and Denver II. The other girl (ID 12), aged 43 months, was not diagnosed with ASD in 2013. Her CARS2 score in 2015 was 42, and she failed the language area of the Denver II and all domains except gross motor of the ASQ. The DSM-IV criteria were used to exclude Rett syndrome and confirm autistic disorder in both girls in 2015 survey. Therefore we diagnosed these two girls as ASD. The first girl had bilateral cataracts (for which she had had surgery), microphthalmia, strabismus, and pigmentary retinopathy; the second girl had bilateral hyperopia and pigmentary retinopathy. Both of them had bilateral moderate or greater hearing impairment. The first girl liked playing alone and watching lamps, fans, fingers, and red toys. The second girl liked playing alone, watching her hands and fingers turning, putting certain toys into her mouth, and looking up at the sky.

### Congenital heart findings

Fifteen in 21 (71.4%) had congenital heart disease at enrollment. Seven had patent ductus arteriosus, one of which combined pulmonary hypertension, two had patent ductus arteriosus and atrial septal defect accompanied by pulmonary hypertension, one had ventricular septal defect with pulmonary hypertension, three had patent ductus arteriosus and pulmonary stenosis, two of which developed pulmonary hypertension, and one had atrial septal defect. Nine of twelve cases with patent ductus arteriosus had catheter occlusion therapy prior to our examination in October 2013. No one had cardiac surgery before and during the study period.

## Discussion

A large number of CRS cases emerged during the 1963–1965 rubella epidemic in the United States and the consequent developmental and sensory difficulties among patients were extensively studied and reported^4^,^5^,^16^,^17^,^18^,^19^. Since then, very few studies^20^ have been done to characterize these developmental and sensory problems, except for those involving a small number of CRS cases^21^. The studies in the 1960s and 1970s, however, were based on old psychiatric and psychological diagnoses and Kanner’s original definition of autism^5^,^22^. This is the first prospective study to describe developmental and sensory difficulties among children with CRS using recently established and current standardized methods.

### Sensory defects

Among 21 patients with CRS evaluated using the AABR test, 13 (62%) had hearing impairment; among these, 10 had moderate or greater bilateral hearing impairment, which would affect their language development in the absence of any appropriate aids or education. Our results were consistent with previous reports that hearing impairment is a common complication of CRS (66–73% of cases) and is generally bilateral and sensorineural^5^,^23^,^24^. We could not conduct further confirmatory assessments owing to unavailability of diagnostic audiological testing facilities in the study area. Instead, we performed the AABR twice or more; those children who were evaluated in both 2013 and 2015 had the same results in both years. We also obtained detailed information from caregivers on the children’s activities of daily living, to confirm the accuracy of our assessment. In 2013, four participants with bilateral hearing impairment based on AABR had otitis media with effusion (unilateral in two and bilateral in two); however, none of them had middle ear effusion in 2015. Therefore, we consider that the influence of effusion upon the AABR results to be minimal in our cases. Among 21 patients with CRS examined by an ophthalmologist, 11 (52%) had abnormal ophthalmological findings and seven (33%) had functional problems other than pigmentary retinopathy, which usually does not affect visual acuity^23^. Previous studies^23^,^24^ have showed that 78–88% of patients with CRS had ocular complications. The most common finding was pigmentary retinopathy (50–60%) followed by cataracts (27–34%), nystagmus, strabismus, microphthalmia, amblyopia, and glaucoma. The prevalence of ophthalmological disorders in our study was slightly lower than in previous studies. These types of sensory defects could impose a great burden on quality of life, affect neuropsychological development, and make developmental assessment and intervention difficult.

### Characterization of developmental status

All children (except for one) who were evaluated using developmental screening tests, had an “abnormal” score in at least one ASQ domain or a “suspect” score in at least one area of the Denver II. The communication domain in the ASQ and language area of the Denver II were the most frequently affected in 2013; the proportions of children with impaired problem-solving and personal–social skills also increased in 2015 (Table 1 and Fig. 2). This could be explained by the high incidence of hearing impairment (Fig. 3) and ASD (Tables 1 and 3) among study participants, both of which can result in language and communication disorders^17^,^19^. Children with a communication or language disorder could have more difficulty with learning social skills with age than children of the same age in the general population. Twelve participants (71%) failed in two or more ASQ domains in 2013 and could be described as having global developmental delay. This is defined as a significant delay in two or more of the following developmental domains: gross/fine motor, speech/language, cognition, social/personal, and activities of daily living^25^. In 12 children tested using the same version of the ASQ in 2013, total ASQ scores ranged broadly from 265 to 0, indicating wide variation in the levels of severity (Fig. 3). In a previous study by Chess^5^, children with congenital rubella had overlapping signs and symptoms such as unspecified, borderline, mild, moderate, severe, or profound intellectual disability (37%); hard signs (44%) of physical neurological defects such as spasticity; and soft signs (24%), such as clumsiness of gait. Eighty-six (95%) children with intellectual disabilities had hearing and/or visual defects simultaneously.

Our findings of multiple developmental difficulties with various levels of severity and high prevalence of sensory complications and communication or language problems were consistent with those of previous studies, even though the diagnostic procedures and definitions of disorders were not identical. An additional problem demonstrated in this study was impaired problem-solving and personal–social skills that become more evident with age.

The ASQ and Denver II used in this study are not confirmatory tests but rather screening tools for developmental difficulties; therefore, they may not be appropriate for identifying developmental concerns in young children with bilateral sensorineural hearing loss^26^, which was very common among our patients. It was difficult in our setting to know whether these children actually had developmental delays in areas of the ASQ and Denver II or whether their hearing or visual impairments led to artificially deflated scores on these measures. Even so, these tests can provide an organized clinical overview of a child’s overall development, which can serve to alert practitioners to potential developmental problems without the need for special training of examiners. These measures should be a first step in the developmental assessment of children with suspected CRS in situations where the availability of a specialist is limited.

### Autism spectrum disorder

In our 2013 study, 7 of 17 children (41%) failed on the M-CHAT and 2 of them (12%) met the DSM-IV criteria^27^ for autistic disorder, which is considered part of ASD according to the DSM-5^28^. Two of the 13 (15%) participants tested by CARS2 in 2015 were diagnosed as having severe ASD, and they also met the DSM-IV criteria for autistic disorder. Thus, 12–15% of the evaluated children with CRS could have been diagnosed as having ASD. This incidence was higher than in a previous CRS study^5^ that reported a 7.4% prevalence of autism and “partial syndrome of autism” by Kanner’s classical criteria^22^.

There were a substantial number of children with CRS who failed on the M-CHAT in our study. However, a combination of sensory and/or other impairments could have increased false positive results because the M-CHAT has 23 items, including 6 that require an intact motor system, 13 requiring visual competence, and 4 requiring intact hearing^29^. Indeed, among the 7 cases with positive screening results, hearing, vision, gross and fine motor impairment were found in 6, 5, 5 and 6 cases, respectively, and 5 cases, including two diagnosed with ASD, had all impairments in combination. There are no approved instruments for making a diagnosis of autism in a child who is hearing impaired. Even common diagnostic tests like the Autistic Diagnostic Observation Schedule include a proviso that it is inappropriate for children who are deaf^30^. Therefore, it is difficult to determine whether the high M-CHAT scores among our study participants indicated a high prevalence of ASD or were the consequence of a combination of hearing and other impairments^31^.

On the other hand, one study found that ASD was more common among children who also have impaired vision or hearing loss compared with an overall population of 8-year-old children in the metropolitan area of Atlanta^32^. Several other studies have proposed rubella as a possible risk factor for co-occurrence of ASD and hearing loss^5^,^33^ or visual impairment^5^,^34^.

In our second developmental assessment in 2015, we performed the CARS2 test, a diagnostic test for ASD. Whereas assessing ASD in children with sensory impairment is difficult using any kind of tool, diagnosis could be more reliable with the use of a diagnostic test and repeating the test once a child was older. More detailed diagnostic examinations and careful follow-up, as well as appropriate interventions including hearing aids, ophthalmological surgeries, eyeglasses, or other training to overcome combined impairments, are required for more precise assessment of ASD among CRS patients^31^.

### Study limitations

We could not conduct more detailed otological and developmental evaluations because we lacked specific equipment and had limited time. The validity and reliability of Vietnamese versions of the ASQ, Denver II, M-CHAT, and CARS2 have not been precisely evaluated, although these screening tests are commonly used in clinical settings in Vietnam. We could not perform statistical tests to compare results between 2013 and 2015 because of limited number of the enrollments.

## Conclusions

The prevalence of global developmental delay and ASD is high among children with CRS. Even though proper assessment is very challenging owing to the presence of combined sensory impairments, thorough and regular assessments as well as timely intervention will be beneficial.

## Methods

### Study setting and period

The study site was Khanh Hoa Province in south-central Vietnam, with a population of 1.15 million in 2009^35^. CRS surveillance was conducted from September 2011 through October 2015 at Khanh Hoa General Hospital (KHGH), the largest and only referral hospital in the province. We followed up the enrolled CRS patients by inviting them to KHGH every 3 months for a general developmental check-up and cardiac examination^9^. Patients were also asked to visit KHGH for ophthalmological and otological examinations in October 2013 and October 2015 and for developmental assessment in December 2013 and July 2015.

### Study participants and case definition

We targeted all neonates and infants under 12 months of age who were born at or referred to KHGH with one or more manifestations suggesting CRS, including: (A) congenital heart disease, cataract(s), glaucoma, or suspected hearing impairment; and (B) purpura, jaundice, hepatosplenomegaly, meningoencephalitis, developmental delay, or microcephaly. The CRS cases were classified into confirmed, probable, and suspected cases according to the following case definitions (the Centers for Disease Control and Prevention^36^): a confirmed case is one with any clinical manifestations of CRS confirmed on a laboratory test; a probable case is one that is not laboratory confirmed but includes either 2 of the clinical signs listed in group (A) or 1 of the clinical signs listed in group (A) and 1 of the clinical signs listed in group (B) with no evidence of any other etiology; and a suspected case is one with some compatible clinical symptoms but that does not meet the criteria for a probable case. The cases were laboratory confirmed based on the detection of either rubella-specific immunoglobulin M antibodies on admission or rubella-specific immunoglobulin G antibodies after 6 months of age. Laboratory test used for confirmation was described previously^9^.

### Otological and ophthalmological examinations

Automated auditory brainstem response (AABR) (Echo-Screen MAAS, Nippon-Koden, Japan) was used to screen for hearing impairment among the children with CRS, first at enrollment and again when children visited KHGH in October 2013 and October 2015. With the children under sedation, stimuli were presented at 35 and 45 decibels, corresponding to normal hearing levels. In both 2013 and 2015, external auditory canals and tympanic membranes were examined by an otolaryngologist (K.K.) with an otoscope before AABR testing; earwax was removed if present. The children also underwent ophthalmic examination by an ophthalmologist (M.U.) and a certified orthoptist on the same day as otological examination. Eye examinations included strabismus examination, slit-lamp examination of the anterior segment, measurement of refraction using a handheld auto refractometer (Retinomax 2; Righton, Japan), intraocular pressure measurement using a rebound tonometer (Icare PRO, Icare Finland, Finland), and posterior segment examination by means of indirect ophthalmoscopy through dilated pupils. Cycloplegic refraction was not measured. Refraction was not measured in participants who had untreated cataracts or aphakia after cataract surgery. Myopia and hyperopia were defined as diopters <−3.0 and >+3.0, respectively. The CADET test of visual acuity^37^, a method based on matching drawings of objects, was performed in 2015, with the support of a local ophthalmologist. We defined abnormal ophthalmological findings as functional ophthalmological impairment and excluded pigmentary retinopathy.

### Developmental assessment

Developmental difficulties were screened or assessed using the Ages and Stages Questionnaire, Second Edition (ASQ)^10^,^11^ and Denver Developmental Screening Test II (Denver II)^12^,^13^ in both 2013 and 2015. Children who failed the ASQ or Denver II were assessed using the Modified Checklist for Autism in Toddlers (M-CHAT)^14^ in 2013 and Childhood Autism Rating Scale, Second Edition (CARS2)^15^ in 2015, all of which were translated into Vietnamese. All testing was performed under the supervision of an experienced Vietnamese paediatric neurologist (G.T.H.N.). The reason we used M-CHAT in 2013 and CARS2 in 2015 was that M-CHAT should be used for toddlers between 16 and 30 months of age and CARS2 can generally be used in children between 2 and 6 years of age.

The ASQ is a parent-administered, structured questionnaire that includes questions in five domains of child development: communication, gross motor skills, fine motor skills, problem-solving skills, and personal–social skills. The scores for each domain are summed, and if the score for any one of the five domains is under the cut-off point (“abnormal” score), the child is considered to have “failed” the screening. The questionnaire closest to the child’s chronological age was administered. The Denver II assesses the child’s performance on various age-appropriate tasks, including 125 items in four areas: personal–social, fine motor–adaptive, language, and gross motor. Each test item was scored as either pass or fail. For each category in the overall assessment, participants were considered to be “suspect” if they failed two or more test items that 75–90% of children their age could pass or if they failed one or more test items that more than 90% of children younger than them could pass. Otherwise, each child’s development was considered to be “normal”.

M-CHAT is designed to screen for autism spectrum disorders (ASD) in toddlers. Parents or caregivers are asked to report on 23 behaviours related to sensory abnormalities, motor abnormalities, social interchange, early joint attention/theory of mind, early language and communication. A positive (“failing”) screening result on M-CHAT is defined as failing two or more “critical” items (items 2, 7, 9, 13, 14 and 15) or any three or more in total; see Robins *et al*., appendix^14^ for details. CARS2 is designed as a clinical rating scale for a trained clinician to rate a child on 15 items indicative of ASD after direct observation of the child, using a 4-point response scale for each item, so as to distinguish children with ASD from developmentally handicapped children who are not autistic. Rating values for all items are summed to produce a total raw score, which indicates the corresponding severity level: 15–29.5, minimal to no symptoms of ASD; 30–36.5, mild to moderate symptoms of ASD; 37 and higher, severe symptoms of ASD. The examiner also takes notes concerning the behaviours relevant to that item while observing the child^15^. We used the total raw score to diagnose ASD (30 and higher) and assess severity (as above) and used the notes to describe participants diagnosed with ASD. Those children with CRS who failed the M-CHAT were further examined by an experienced paediatric neurologist, who focused on the child’s eye contact, response to non-verbal approach, and the presence of any abnormal behaviour or disinterest. A diagnosis of autistic disorder was made according to criteria of the Diagnostic and Statistical Manual of Mental Disorders (DSM-IV)^27^. For those diagnosed as having ASD by CARS2, we further used the DSM-IV criteria for autistic disorder and Rett syndrome, for confirmation and differential diagnosis. We used the DSM-IV instead of the DSM-5 in this study since the latter is not available in Vietnamese. However, we used the concept of “autism spectrum disorder” (ASD) in the DSM-5, which includes “autistic disorder” in the DSM-IV, for our results analyses and discussion^28^.

### Data analysis

Demographic characteristics and symptoms were described using simple tabulation. Means and standard deviations of scores for each domain of ASQ were calculated using data of 1,494 Vietnamese participants in a previous birth cohort 2-year follow-up study^38^ and used as standard scores for the 24-months ASQ in Vietnamese children (manuscript in preparation). All statistical analyses were conducted using Stata version 12.0 software (Stata Corp LP, College Station, TX, USA).

### Ethics

The Institutional Review Boards of the National Institute of Hygiene and Epidemiology, Hanoi and the Institute of Tropical Medicine, Nagasaki University approved this study. Anonymized data were used for the analyses. Informed consent was obtained from all parents or guardians before conducting the examinations. All methods were performed in accordance with the relevant guidelines and regulations.

## Additional Information

**How to cite this article**: Toizumi, M. *et al*. Sensory defects and developmental delay among children with congenital rubella syndrome. *Sci. Rep.* **7**, 46483; doi: 10.1038/srep46483 (2017).

**Publisher's note:** Springer Nature remains neutral with regard to jurisdictional claims in published maps and institutional affiliations.

## Figures and Tables

**Table 1:** Developmental and sensory assessment of children with congenital rubella syndrome.

ID		1	2	3	4	5	6	7	8	9	10	11	12	13	14	15	16	17	18	19	20	21
Sex		F	F	F	F	M	F	M	M	F	F	M	F	M	M	F	F	F	F	M	M	M
**Developmental milestone before exams**		−	−	+	+	+	+	+	+	+	+	+	+	+	+	+	−	−	+	−	+	−
**Development in 2**0**13**	age (month)	24	24	26	26	24	25	24	25	24	24	22	24	28	21	23	19	10				
**ASQ**																						
	communication	+	+	+	+	+	+	+	+	+	−	+	+	+	+	+	−	−				
	gross motor	−	−	+	+	−	−	−	+	+	−	+	+	+	+	+	−	−				
	fine motor	−	+	+	+	−	−	−	−	+	+	+	+	−	+	+	−	−				
	problem solving	−	−	+	+	+	−	−	−	+	−	+	−	−	+	−	−	−				
	personal-social	−	−	+	+	−	−	+	+	+	−	+	−	−	+	−	−	−				
**Denver II**																						
	individual-social	−	−	+	+	−	−	−	+	+	−	+	−	−	+	−	−	−				
	fine motor-adoption	−	−	+	+	−	−	−	−	+	−	+	−	−	+	−	−	−				
	language	−	+	+	+	+	−	+	−	+	−	+	+	+	+	+	+	+				
	gross motor	−	−	+	+	−	−	−	−	+	−	+	−	−	+	+	−	−				
**M**-**CHAT**		−	+	+	−	−	−	+	−	+	−	+	+	−	+	−	−	−				
**Autism spectrum disorder**			^a^	−				^a^		+		+	^a^		^a^							
**Development in 2**0**15**	age (month)		43	45		43		43	45	44			43		41	42	38		45	44		36
**ASQ**																						
	communication		−	+		+		+	+	+			+		+	+	−		+	−		+
	gross motor		−	+		−		−	−	+			−		+	+	−		+	−		+
	fine motor		−	+		−		+	−	+			+		+	+	−		+	−		+
	problem solving		−	+		+		+	−	+			+		+	+	−		+	−		+
	personal-social		−	+		+		+	−	+			+		+	+	−		+	−		+
**Denver II**																						
	individual-social		−	+		+		−	−	+			−		+	−	−		+	−		+
	fine motor-adoption		−	+		−		−	−	+			−		+	−	−		+	−		−
	language		+	+		+		+	−	+			+		+	+	−		+	−		−
	gross motor		−	+		+		−	−	+			−		+	+	−		+	−		−
**CARS2**			18	27.5		23.5		25	16.5	40.5			42		23.5	28	16.5		27	NA		20.5
**Eye and ear in 2**0**13**	age (month)	22	23	24	24	22	23	22	24	23	23	21	22	26	20	21	17	8	24	23	22	15
**Eye and ear in 2**0**15**	age (month)		47	48		47		46	48	47	47	45	46		44	45	41		48	47	46	39
**Cases examined in both years** (*)			*	*		*		*	*	*	*	*	*		*	*	*		*	*	*	*
**Ear**	Intervention					HA														CI		
AABR		−	−	+	−	+	^b^	+	−	+	−	+	+	left	+	+	−	−	left	^c^	+	−
Otitis media with effusion in 2013		−	−	−	−	−	−	+	−	left	−	−	+	−	−	−	+	−	−	−	right	−
**Eye**	Intervention						O			O, G						O, G						
Pigmentary retinitis		−	+	+	−	+	+	−	+	+	−	−	+	−	−	+	−	−	+	−	+	−
Cataract		−	−	−	−	−	left	−	−	+	−	+	−	−	−	+	−	−	−	−	−	−
Microphthalmia		−	−	−	−	−	left	−	−	+	−	+	−	−	−	+	−	−	−	−	−	−
Strabismus		−	−	+ in	−	−	left	−	−	+	−	+	−	−	−	+	−	−	−	−	+ in	−
Nystagmus		−	−	−	−	−	−	−	−	−	−	+	−	−	−	+	−	−	−	−	−	−
Myopia		−	−	+	−	−	+^d^	−	−	^e^	−	^f^	−	−	−	^e^	−	−	−	−	−	−
Hyperopia		−	−	−	−	−	+^d^	−	−	^e^	−	^f^	+	−	−	^e^	−	−	−	−	−	−
**Vision test in 2**0**15**			1.0/1.0	NC		NC		NC	1.0/1.0	NC	1.0/1.0		NC		NC	NC	1.0/1.0		NC	1.0/1.0	NC	0.6/0.7
**Cardiac**		+	+	−	+	+	−	+	+	−	+	+	+	−	+	−	+	+	−	+	+	
**Category**		C	C	S	C	C	C	C	C	C	C	C	P	P	C	P	C	C	C	C	P	

NA; not examined.

<Development>

Milestone: +Developmental delay with not holding the head up at or over 6 months of age and/or sitting up at or over 10 months of age by interview with a caregiver.

The Ages and Stages Questionnaire (ASQ): +“Abnormal” with score for each domain below the cut-off, −“Normal” with score over the cut’-off, no mark; Not examined.

Denver II: +“Suspected” with two or more cautions and/or one or more delays, −“Normal” with no delays and a maximum of one caution, no mark; Not examined.

Modified Checklist for Autism in Toddlers (M-CHAT): +Positive with failure of any 3 of the 23 total items or 2 of the 6 critical items, −Not positive, no mark; Not examined.

Autism spectrum disorder: +Autism spectrum disorder diagnosed by Diagnositic and Statistical Manual of Mental Disorders-IV criteria for autistic disorder.

−No autism spectrum disorder diagnosed by Diagnositic and Statistical Manual of Mental Disorders-IV criteria for autistic disorder.

^a^No autism spectrum disorder diagnosed by a pediatric neurologist’s examination.

no mark; No autistic spectrum disorder with negative result of M-CHAT.

CARS2; total Raw score in Childhood Autism Rating Scale, second edition

<Ear>

Intervension: HA; hearing aid, C; cochlear implant

Automated auditory brainstem response (AABR): +Refer at 45dBnHL in both ears, −Pass at 35dBnHL in both ears, left; Pass at 35dBnHL in right ear and refer at 45 dBnHL in left ear.

^b^Both pass at 45dBnHL and refer at 35dBnHL, ^c^Cochlear implant for both ears and AABR was not performed

Otitis media with effusion: −No otitis media with effusion or retraction of tympanic membrane in 2013, +/left/right; Otitis media with effusion or retraction of tympanic membrane

in both/left/right ear(s) in 2013 and all were cured in 2015.

<Eye>

Intervension: O; operation for cataract, G; glasses after operation

+With the finding, −Without the finding, left; Unilateral finding in left eye, in; intermittent external strabismus

Vision test: 1.0/1.0; 1.0 in both eyes by vision test, NC; child could not cooperate

^d^Right eye had myopia and left eye had hyperopia with intraocular lens after cataract surgery, ^e^Aphakia after cataract surgery, ^f^Refrection unmeasurable due to cataract.

**<**Cardiac>

+Cardiac defects including patent ductus arteriosus, atrial septal defect, pulmonary artery stenosis and pulmonary hypertention, −No cardiac defect.

<Category>

C; confirmed CRS, P; probable CRS, S; suspected CRS.

**Table 2:** Ophthalmological complications with congenital rubella syndrome in 2013 and 2015 (n = 21).

(n = 21)	n (%)
pigmentary retinopathy	10 (48)
strabismus^a^	6 (29)
cataract	4 (19)
microphthalmia	4 (19)
nystagmus	2 (10)
myopia^b^ (n = 18)^c^	2 (11)
hyperopia^b^ (n = 18)^c^	2 (11)

^a^Two cases had intermittent external strabismus.

^b^Including one case who had one eye with myopia and the other with hyperopia with intraocular lens after cataract surgery.

^c^Refrection was not measured for 3 cases with bilateral cataract or aphakia after surgery and they were not included into number for calculation of proportion of myopia or hyperopia.

**Table 3:** M-CHAT scores, hearing, vision, and gross/fine motor impairments, and 2015 CARS scores among M-CHAT positive cases in 2013.

ID	2	3	7	9	11	12	14
Age (month)	24	26	24	24	22	24	21
Sex	F	F	M	F	M	F	M
M-CHAT	2	13	7	17	14	3	9
No. of failed “critical” items	2	5	3	4	4	2	4
No. of failed other items	0	8	4	13	9	1	5
Hearing	−	+	+	+	+	+	+
Eye	PR	+	PR	+	+	+	+
Gross motor	−	+	−	+	+	+	+
Fine motor	+	+	−	+	+	+	+
diagnosed as ASD in 2013	^a^	−	^a^	+	+	^a^	^a^
CARS score in 2015	18	27.5	25	40.5	NA	42	23.5

Age: Age at the examination, Sex: F; Femaile, M; Male

M-CHAT: Total number of failed items of Modified Checklist for Autism in Toddlers

Hearing: +Suspicious of bilateral hearing impairment above 45dBnHL, −No suspicious hearing impairment.

Eye: +Ophthalmological disorders in function including cataract(s), myopia, strabismus, nystagmus, microphthalmia and amblyopia,

−No ophthalmological disorders, PR; Pigmentary retinopathy.

Gross/Fine motor: ‘+Scored under the cut-off point in gross/fine motor domain of the Ages and Stages Questionnaire.

−Scored over the cut-off point in gross/fine motor domain of the Ages and Stages Questionnaire.

Autism spectrum disorder (ASD): +ASD diagnosed by Diagnositic and Statistical Manual of Mental Disorders-IV criteria for autistic disorder.

−No ASD diagnosed by Diagnositic and Statistical Manual of Mental Disorders-IV criteria for autistic disorder.

^a^No ASD diagnosed by a pediatric neurologist’s examination.
